# Impact of exosome therapy on pancreatic cancer and its progression

**DOI:** 10.1007/s12032-023-02101-x

**Published:** 2023-07-05

**Authors:** Mohamed El-Tanani, Hamdi Nsairat, Ismail I. Matalka, Alaa A. A. Aljabali, Vijay Mishra, Yachana Mishra, Gowhar A. Naikoo, Sai Raghuveer Chava, Nitin B. Charbe, Murtaza M. Tambuwala

**Affiliations:** 1 Pharmacological and Diagnostic Research Center, Faculty of Pharmacy, Al–Ahliyya Amman University, Amman, 19328 Jordan; 2 Institute of Cancer Therapeutics, University of Bradford, Bradford, BD7 1DP West Yorkshire UK; 3 Ras Al Khaimah Medical and Health Sciences University, Ras Al Khaimah, UAE; 4 Department of Pathology and Microbiology, Faculty of Medicine, Jordan University of Science and Technology, Irbid, 22110 Jordan; 5 Department of Pharmaceutics and Pharmaceutical Technology, Faculty of Pharmacy, Yarmouk University, P.O. Box 566, Irbid, 21163 Jordan; 6 School of Pharmaceutical Sciences, Lovely Professional University, Phagwara, 144411 India; 7 Department of Zoology, School of Bioengineering and Biosciences, Lovely Professional University, Phagwara, 144411 India; 8 Department of Mathematics and Sciences, College of Arts and Applied Sciences, Dhofar University, PC 211, Salalah, Oman; 9 Department of Chemistry, Texas A&M University, Kingsville, TX USA; 10 Department of Pharmaceutics, College of Pharmacy, Center for Pharmacometrics and Systems Pharmacology, University of Florida, Orlando, FL USA; 11 Lincoln Medical School, University of Lincoln, Brayford Pool Campus, Lincoln, LN6 7TS UK

**Keywords:** Exosomes, Pancreatic cancer, Intercellular communication, Biomarkers, Folfirinox, Gemcitabine

## Abstract

Pancreatic cancer, one of the most aggressive tumors, has a dismal prognosis because of the low rates of early identification, fast progression, difficulties following surgery, and the ineffectiveness of current oncologic therapies. There are no imaging techniques or biomarkers that can accurately identify, categorize, or predict the biological behavior of this tumor. Exosomes are extracellular vesicles that play a crucial rule in the progression, metastasis, and chemoresistance of pancreatic cancer. They have been verified to be potential biomarkers for pancreatic cancer management. Studying the role of exosomes in pancreatic cancer is substantial. Exosomes are secreted by most eukaryotic cells and participated in intercellular communication. The components of exosomes, including proteins, DNA, mRNA, microRNA, long non-coding RNA, circular RNA, etc., play a crucial role in regulating tumor growth, metastasis, and angiogenesis in the process of cancer development, and can be used as a prognostic marker and/or grading basis for tumor patients. Hereby, in this concise review, we intend to summarize exosomes components and isolation, exosome secretion, function, importance of exosomes in the progression of pancreatic cancer and exosomal miRNAs as possible pancreatic cancer biomarkers. Finally, the application potential of exosomes in the treatment of pancreatic cancer**,** which provides theoretical supports for using exosomes to serve precise tumor treatment in the clinic, will be discussed.

## Introduction

Pancreatic cancer is the sixth worst cancer in the world, according to statistics [1]. The most prevalent form of the disease is pancreatic ductal adenocarcinoma (PDAC). If pancreatic surgery is performed and the cancer has not spread, pancreatic cancer patients diagnosed in the United Kingdom have a 7–25% probability of surviving five years [2]. Despite the fact that surgery has been shown to prolong life, only 20% of PDAC patients with respectable tumors may undergo it [1]. According to Cancer Research UK, 9263 deaths are believed to have occurred in 2016 alone. ‘In a great majority of cases, the disease has already advanced at the time of diagnosis. In addition, patients do not respond well to the available treatments, and there is a high chance of metastasis, which is difficult to detect on scans [1]. Surgery and adjuvant chemotherapy are currently the sole treatment options for pancreatic cancer. Folfirinox and Gemcitabine are the chemotherapeutic agents administered [3–5]. As with all chemotherapies, chemoresistance and severe toxicity are common side effects of chemotherapeutic treatments [6, 7]. In spite of the availability of treatment, there has been little increase in PDAC survival rates over the past four decades. [8]. Exosomes are a form of extracellular vesicle that are currently the subject of extensive research. The significance of exosomes released by pancreatic cancer cells in metastasis and treatment will be surveyed in this review.

## Components and isolation

In 1987, Johnstone RM et al., found the in vitro release of extracellular vesicles from sheep reticulocytes, which led to the first description of exosomes [9]. Exosomes were created as a result of the classification of extracellular vesicle subtypes made possible by future research. A variety of cell types, including dendritic cells, epithelial cells, T and B cells, and cancer cells, secreting exosomes [10]. The cerebrospinal fluid, saliva, urine, and blood all had considerable levels of exosomes, according to an analysis of physiological fluids [10, 11]. Exosomes, distinguishable by their size and the unique protein and lipid rafts on their plasma membrane, have a diameter of 30–100 nm and are heterogeneous despite being discharged from the same cell [10, 12].

Most of the research utilize ultracentrifugation, the conventional technique for obtaining exosomes. Compared to earlier techniques, ultracentrifugation yields more pure exosomal samples, and the preparation consists of exosomes of roughly the same size [13]. Ultracentrifugation damages extracellular vesicles, according to studies on exosomal separation techniques [14]. Richard J. Lobb et al., discovered that ultrafiltration is a more effective technique for isolating exosomes due to its higher exosomal yield and shorter processing time [15].

**Fig. 1:**
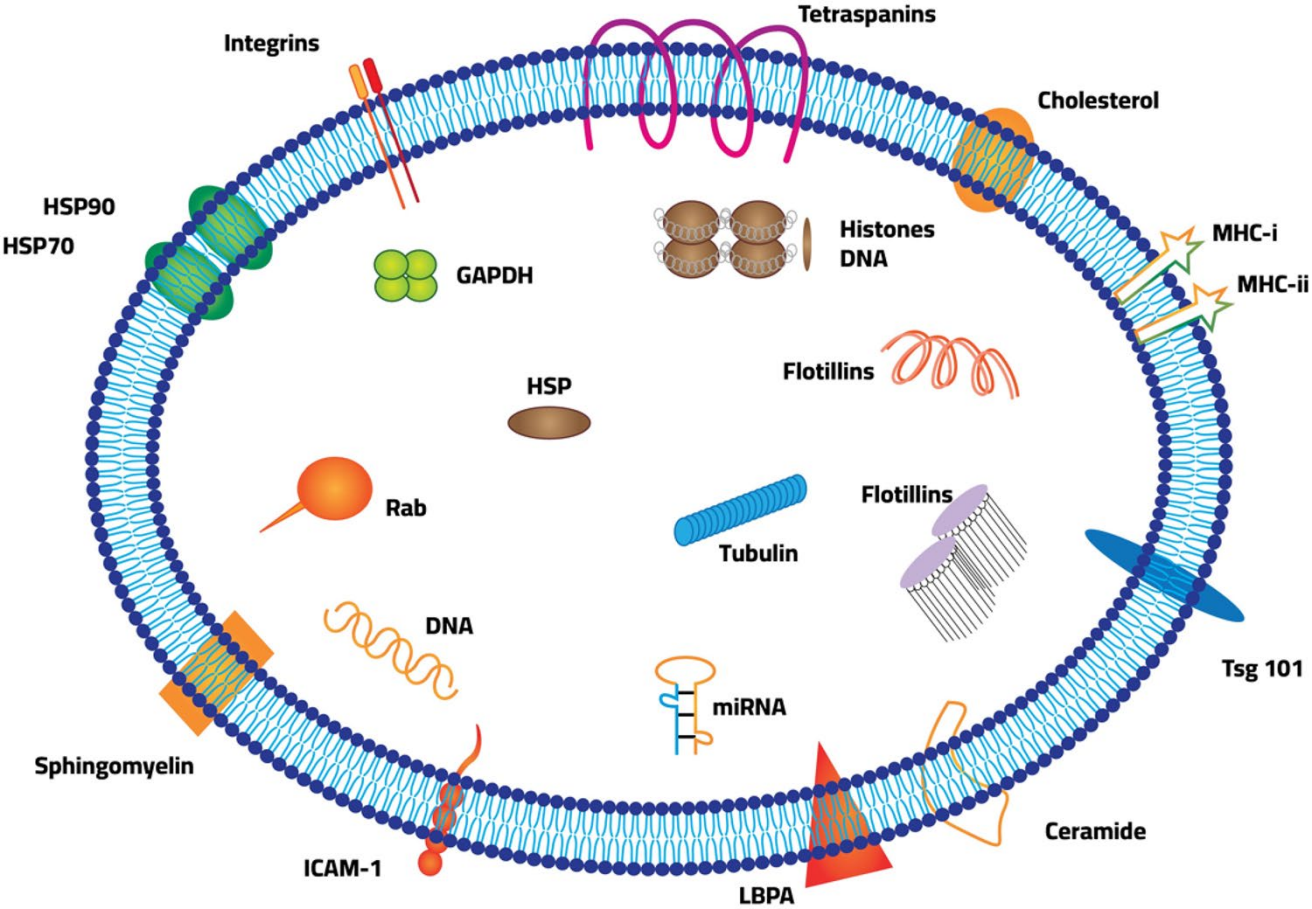
A typical exosomes composition and structure of exosomes

According to the exosome database (http://www.exocarta.org), exosomes comprise 9769 proteins, 3408 messenger RNAs, 2838 microRNAs, and 1116 lipids [16]. Lipids, proteins, DNA, messenger RNA, non-coding RNAs, and proteins can all operate as autocrine or paracrine agents (Fig. 1). They control angiogenesis, metastasis, tumor development, and treatment resistance at the gene level through paracrine action [17, 18].

MVBs create exosomes from membrane transporters, HSPs, and other proteins. It contains microRNA, long non-coding RNA, and circular RNA. Malignancies require these elements. MSCs (mesenchymal stromal cells), CSCs (cancer stem cells), CDEs (cancer-associated fibroblast-derived exosomes), and MVBs (mesenchymal vascular blebs) were identified (multivesicular bodies).

Exosomes function as extracellular vehicles (EVs) that transport proteins, lipids, nucleic acids, mRNAs, miRNAs, and nucleic acids to normal and pathological processes. ESCRT-0 uses ubiquitination to detect exosomal proteins, whereas deubiquitination is essential for ILV sorting. Whether ubiquitination drives proteins into exosomes remains debatable [19]. The following proteins are found within exosomes: (i) Membrane transport and fusion-related proteins such as annexin, Rab-GTPase (Ras-related protein GTPase Rab), and heat shock proteins (HSPs) such as Hsp60, Hsp70, and Hsp90; (ii) Tetraspanins (four-transmembrane cross-linked proteins) such as CD9, CD63, CD81, CD82, CD106, TIMP-1, and TIMP-2 (participating in cytoskeletal construction) [19, 20]. Tetraspanins help exosomes to load specific payloads, and CD9 helps exosomes to carry the metalloproteinase CD10. HSPs prevent tumor apoptosis by folding proteins and regulating proteostasis and proteolysis as molecular chaperones [21]. Due to metabolic, nutrient, and acidity deficiencies, hypoxia, and acidosis, cancer cells express high quantities of Hsp90 [22]. Recent MVB-plasma membrane fusion for exosome functions has been mediated via the deformability of the Hsp90 membrane. Without extracellular Hsp90, a key subtype of Hsp90, exosomes cannot stimulate cellular motility via interacting with stromal cells [23].miRNAs, which are short non-coding RNAs consisting of 20–22 nucleotides, link to the 3′-untranslated region or open reading frames of the target messenger RNA to facilitate post-transcriptional gene silencing. In numerous clinical and physiological processes, they have been widely explored [24]. Exosome microRNAs may act as indicators for cancer staging and prognosis. The elevated levels of exosomal miR-451a, miR-21, and miR-4257 in non-small cell lung cancer patients were associated with tumor formation, recurrence, and a poor prognosis. Plasma EV let-7a-5p is lower in men with a high Gleason score (GS) than in men with a low GS [25]. Using exosomal miR-1290 and miR-375, patients with castration-resistant prostate cancer can predict their overall survival. 80% of those with elevated levels of both miRNAs died after 20 months, compared to 10% of those with normal levels [26]. Sun et al., isolated and analyzed exosomes secreted by cancer stem cells (CSCs) and their parental cells and identified six miRNAs (miR-1246, miR-424-5p, miR-628-5p, miR-1290, miR-675-3p, and miR-573-3p) that were upregulated and five that were downregulated, which may serve as biomarkers for identifying individuals at high risk for developing gastric cancer and for diagnosing gastric [27, 28].miR-21, miR-26, miR-122, and miR-150 are cholangiocarcinoma indicators found in the blood. Hu et al., characterized exosomal miRNAs as diagnostic, predictive, and prognostic indicators for lung cancer [29]. Exosomal miR-126 was elevated in 43 plasma exosomes from 45 non-small cell lung cancer patients and 31 healthy controls, indicating its potential application as a diagnostic biomarker [30]. Lung cancer patients’ sera contained elevated levels of circulating exosomal miR-23a, which is positively related to proangiogenic activity and may be employed as a biomarker [21]. Gemcitabine (GEM) response and radiation reaction can be predicted, respectively, by exosomal miR-222-3p and miR-208a. MiR-9 in triple-negative breast cancer exosomes promoted tumor cell motility and transformed normal fibroblasts (NFs) into cancer-associated fibroblasts (CAFs) by inhibiting E-cadherin expression in NFs [31].

Exosomal microRNAs from mesenchymal stem cells (MSCs) or fibroblasts boost cancer cell proliferation and therapy resistance in multiple myeloma (MM), colorectal, and gastric cancers [32, 33]. lncRNA is a brand-new regulatory RNA that can be precisely packaged into exosomes and regulates tumor development, metastasis, angiogenesis, and TME remodeling [34]. A novel cancer-related lncRNA, lncRNAs-ATB, was overexpressed in lung cancer, colorectal cancer, gastric cancer, and hepatocellular carcinoma (HCC). It enhanced carcinogenesis and growth by binding competitively to miRNA (family miR-200) to promote EMT [35]. CD90 and CSC exosomes may enhance angiogenesis in HUVEC, according to Conigliaro et al*.,* (human umbilical vein endothelial cells) [36]. Endothelial cells were able to consume exosomes and distribute lncRNA H19 to target cells by adhering to CD90 cells and HUVECs and encouraging angiogenesis by generating and releasing vascular endothelial growth factors. LncRNA 57 enhances tumor resistance. The lncRNA [37] UCA1 increased Wnt6 expression and cisplatin resistance in bladder cancer cells. Hence, the UCA1/Wnt6 pathway may be able to prevent bladder cancer [38]. scircRNA. Circular RNA (circRNA), a novel family of endogenous non-coding RNA, is present in all eukaryotic cells. Reverse splicing generates sixty circRNAs containing alternative splices. circRNAs, as endogenous RNA, compete with miRNAs, preventing miRNA from targeting mRNA [39]. In cancer, circular RNAs such as circCCDC66, circHIPK3, circPVT1, and cirs-7 work as competitive inhibitors of microRNA and affect protein function or their own translation. Circ-IARS was raised in both the in situ and metastatic plasma of PC. By reducing miR-122 and ZO-1 and enhancing RhoA and RhoA-GTP, F-actin expression and adhesion, and endothelial permeability, Circ-IARS overexpression boosted tumor invasion and metastasis [40, 41]. Circ-DLEU2 overexpression enhanced PRKACB expression by inhibiting miR-496, which increased leukemia cell proliferation in vitro, halted cell death, and led to the development of acute myeloid leukemia tumors in vivo [42].

Since circRNA expression levels are typically related to clinicopathologic characteristics, these RNAs may serve as diagnostic, prognostic, and predictive biomarkers [43]. CircumCNOT2 was identified in the plasma of patients with primary breast cancer, suggesting that it could be utilized as a biomarker for selecting the optimal course of treatment or for less invasive monitoring of disease development [44]. The expression of circ-KLDHC10 on serum exosomes was used to distinguish colon cancer patients from healthy controls [45].

Exosomes are able to permeate membranes, including the blood–brain barrier, due to their small size; this is a highly favorable property that would allow for deep penetration within the target organ [46]. Exosomal separation and modification techniques require additional study. Because exosomes can be obtained from so many different locations, it might be difficult to determine which is the optimal source [47]. Exosome manufacture at a large scale is difficult due to the inefficacy of existing procedures. Extraction of exosomes from biological fluids and loading of exosomes with therapeutic compounds require additional research, particularly if exosome therapy is to be used for a variety of cancer treatments. Exosomal dose and potential adverse effects of blocking healthy tissue from secreting exosomes must also be evaluated in clinical trials [48–50].

## Exosome secretion

**Fig. 2:**
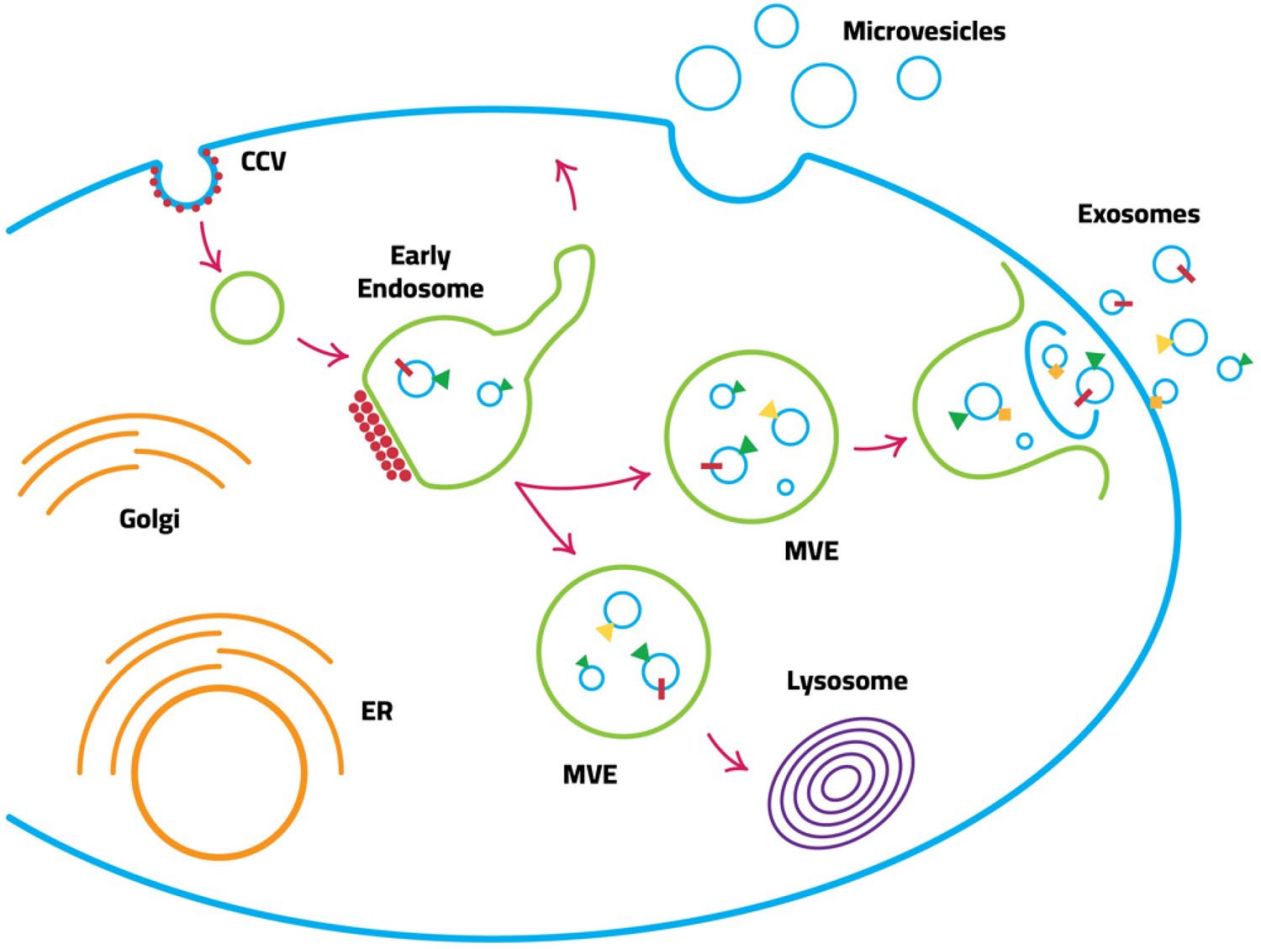
Production and secretion of exosomes

Exosome secretion involves four steps: initiation, endocytosis, production of multivesicular bodies (MVB), and lastly exosome release [68]. Figure 2 depicts the production of exosomes in a schematic format. Several invaginations of the lipid bilayer of a cell form an endosome cell containing MVBs [69]. MVBs accumulate in the endosome lumen, where they are sorted and either lysed or exocytosed into the extracellular milieu [10, 69]. Exosomes are the contents that are released into the extracellular environment. This explains why the orientation of the exosomal lipid bilayer matches that of the initiating cell. Exosome sorting and biogenesis occur in MVBs and include several regulatory mechanisms; hence, this concept will not be researched due to a lack of data [10]. Due to their endosomal origin and the presence of proteins linked with membrane fusion and transport, exosomes can play a role in metastasis [10, 70].

MVBs, internal multivesicular compartments, create exosomes (LEs). Tetraspanins, ysobisphosphatidic acid (LBPA), and ESCRT endosomal sorting complex required for transport) proteins have all been demonstrated to be necessary for the formation of the interior vesicles of MVBs; however, it is still unknown how each of these molecules contributes to exosome biogenesis. Recent research has also demonstrated that lipid ceramide is necessary for exosome secretion, allowing the creation of interior vesicles. Exosome secretion has been linked to a number of Rab proteins, including RAB11, RAB27, and RAB35, which are known to be involved in the movement of vesicles between intracellular compartments. The fusing of MVBs with the plasma membrane, the last step in exosome secretion, most likely involves a complex of SNARE proteins, but the specifics of this complex are unknown.

## Function

Exosomes are believed to be involved in the expulsion of non-functional/excess cellular material, despite a lack of clarity in the scientific literature regarding their precise role and actions [71]. It is believed that this mechanism occurs in cells with minimal degradative activity. Furthermore, proteins on the plasma membrane of exosomes are believed to activate cell receptors to initiate cell signaling pathways [69]. Exosomes are believed to contribute to intracellular communication by influencing the fundamental processes of the recipient cell [72]. Valadi et al., discovered 13 examples of functional genetic material exchange; this function is believed to be critical for PDAC spread and carcinogenesis [69, 73, 74].

### Importance of exosomes in the progression of pancreatic cancer

**Fig. 3:**
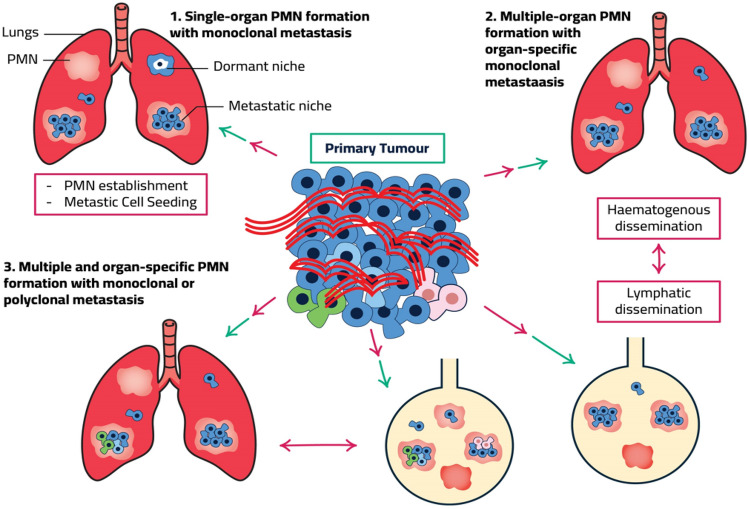
Exosomes impact on pancreatic cancer progression

Metastasis is a dynamic process characterized by the spread of tumor cells from the primary organ to secondary and tertiary sites in the body. The lymphatic and circulatory systems are critical for tumor cell migration. In pancreatic cancer, where metastases to the liver, lungs, and peritoneum are common, therapeutic failures are commonly attributed to metastasis [75]. The “seed and soil” idea, proposed in 1889 by Stephan Paget, describes how cancer cells interact with distant organs to produce a microenvironment that favors the growth and survival of metastatic cells (the seeds) [76]. This milieu is referred to as a pre-metastatic niche (PMN), and it has been hypothesized that exosomes contribute to the establishment of PMNs (see Fig. 3) [77].

1. Pre-metastatic niches are created in target organs by primary tumor-secreted components and EVs (PMNs). PMN that have been infused with a tumor grow and micrometastasize. Without PMN, metastatic colonization fails. In contrast to PMNs, dormant niches encourage CTC slumber rather than metastatic growth. For cancer cells to go into quiescence and dormancy, TSP1, which is deposited around stable microvasculature by stromal cell-expressed TGF and BMP, is crucial. DTC growth may be halted by dormant metastatic organs. 2. Most solid tumors can spread to numerous organs. EVs and tumor-secreted substances can stimulate the growth of PMNs in a variety of tissues and seed metastatic tumor cells. Research into organ-specific seeding is extensive. Organ-specific metastases are brought on by the metastatic colonization of tumor subclones. Metastasis is brought on by lymphatic or hemogenous spread. Sarcomas spread through the blood vessels, whereas epithelial cancers move through the lymphatic system. 3. PMNs are distributed to several organs by primary tumor subclones or secondary metastatic microenvironments. Organ-specific PMNs have the potential to develop polyclonal metastatic lesions that contain several tumor cell subclones.

Costa Silva et al., in a study on pancreatic cancer exosomes and hepatic PMN production, found that 16 Exosomes generated by PDAC tumors in mice were isolated using ultracentrifugation, the industry standard [78]. Using electron microscopy and nanoparticle characterization, the usual size and structure of exosomes were uncovered (100 nm) [13, 78] Due to the substantial overlap in size of extracellular vesicles, exosomal purification methods have proven to be difficult. According to the study's analysis of cell appearance and structure, the sample contained exosomes.

The modification of the extracellular matrix is one of the most crucial phases of PMN development [79]. This study demonstrates that PDAC-derived exosomes promote the formation of a pre-metastatic environment that is conducive to the germination and micrometastasis of tumor cells [78]. Exosomes from PDAC were injected into the portal veins of mice. Kupffer cells specifically absorbed transforming growth factor, initiating the production and signaling pathway (TGF) [78]. This in turn stimulated fibronectin overexpression in hepatic stellate cells. The fibrotic microenvironment attracted macrophages originating from bone marrow, establishing an inflammatory milieu. It is hypothesized that this immune dysregulation will occur in PMN tissues, hence, creating a favorable environment for the implantation of pancreatic cancer cells [78]. In their review, Massoumi et al., found raised TGF levels and elevated fibronectin deposition in the peritoneal tissue of PDAC patients [75].

Because to the parallels between these findings and those found in the liver, the concept of a peritoneal PMN was proposed [80]. Paget's theory of PMNs is supported by the evidence, although it is crucial to note that the conclusions in this review have not yet been published and that additional study is required to completely comprehend the molecular components essential for peritoneal PMN production [75, 81]. Organotropism or organ-specific metastasis is an alternative explanation for Paget's concept; tumor-released exosomes can be thought of as “fertilizers” since they stimulate the colonization of cancer cells in the PMN [82, 83].

Several studies have demonstrated exosomes’ significance in PMN synthesis. Even though this review focuses on pancreatic cancer metastasis, the fundamental concepts behind PMN development apply to all cancers [82]. Malignant exosomes, for instance, enhance the overexpression of inflammatory markers, drive angiogenesis, and influence immune function. The spread of cancer is a consequence of these channels. Exosomes and PMN synthesis remain unknown. Given that exosomes remain in circulation, the excision of the underlying tumor does have any effect on PMN production [82, 84].

The well-known cytokine macrophage migration inhibitory factor (MIF) causes inflammation and modifies liver fibrosis [85]. Exosomes contain the MIF protein, which stimulates TGF secretion. Exosomal MIF reduction decreased fibronectin production and attracted fewer macrophages, demonstrating that MIF is necessary for PMN formation and, ultimately, liver metastasis [78]. Costa-Silva's analysis of the clinical significance of these data revealed that individuals with stage 1 PDAC had increased exosomal MIF concentrations in their blood and the cancer has not metastasized. On the other hand, MIF levels were lower in PDAC patients with liver metastases [78]. It is likely that MIF might be used as a predictive tool to enable the prediction of metastasis in high-risk patients, given that MIF-dependent pathways play a crucial role in the earliest stages of metastasis [78, 86]. Funamizu et al. discovered a correlation between MIF overexpression and a decreased survival rate and rapid disease development in PDAC patients, and MIF has been suggested as a potential target for PDAC therapy [87]. Further research is required to determine how MIF affects PMN production and whether it may be utilized to diagnose malignancies other than pancreatic cancer [78].

Because the findings are so significant and the information has shed light on how exosomes plan metastasis, it is imperative to note that all of the review publications included in this study have credited the work of Costa Silva et al., [78]. The clinical significance of MIF in relation to the production of hepatic PMNs has been provided with new research pathways as a result of this work [78].

### Exosome-derived miRNAs as possible pancreatic cancer biomarkers

miRNA is becoming increasingly important. Precursor transcripts make 20–23 nucleotide microRNA that can cause cancer [88]. Cancer may have reduced the amounts of oncogene-targeting microRNAs. When miRNA is placed inside an EV, its half-life is extended [89]. MicroRNA produced by EV outperformed cell-free blood indicators [90].

MiR-192-5p may prevent epithelial cells from transforming into mesenchymal cells by inhibiting ZEB2. Individuals with PDAC and chronic pancreatitis were identified from healthy controls by the expression level of miR-192-5p in exosomes and tissue. CA 19–9 extracted from tissue or exosomes cannot distinguish PDAC from chronic pancreatitis [91].

PDAC patients have greater blood levels of miR-200b/200c than typical. MiR-200b/200b from total and EpCAM + serum exosomes, as well as an increase in CA 19–9, revealed the presence of PDAC with 97% sensitivity. Higher total serum exosome miR-200c levels were linked to a shorter lifetime [92]. Patients with pancreatic cancer and chronic pancreatitis had higher miR-200b/200 concentrations than healthy people [93, 94].

PDAC patients were compared to all other groups using the exosomal surface antigen panel PaCIC such as CD44v6, Tspan8, EpCAM, MET, and CD104 and serum exosomal miRNA indicators miR-1246, miR-4644, miR-3976, and miR-4306. Thus, both sensitivity and specificity improved [1, 95].

Que et al. discovered that PDAC blood samples contained higher levels of exosome-derived miR-17-5p than blood from chronic pancreatitis or healthy persons [96]. There was no correlation between PDAC spreading to other areas of the body and PDAC stages, tumor stages, or differentiation [97].

### Using exosomes to treat pancreatic cancer

The therapy of therapeutic resistance, immunological suppression, and angiogenesis with exosomes has been utilized [75]. Several studies have utilized exosomes to treat pancreatic cancer. Exosomes as drug transporters, as therapeutic targets, and immunotherapy [98].

Exosomes have been utilized in numerous studies as delivery methods, and the oncogenic KRAS mutation is prevalent in pancreatic cancer [99]. Kamerker et al., evaluated the effect of modified exosomes, or iExosomes, on the mutant KRAS gene. Short hairpin RNA (siRNA) that targets oncogenic KRAS is delivered by modified exosomes [99]. CD47, an integrin-associated transmembrane protein of which role is to suppress cellular phagocytosis, was present on the exosomal membrane. CD47 on tumor cells sends a “don't eat me” signal when it binds to the inhibitory receptor signal protein (SIRP). Exosomal CD47 binding to SIRP, which extended exosome half-life by blocking phagocytosis, demonstrates exosomes' ability to avoid the innate immune response [100, 101]. Furthermore, CD47 boosted micropinocytosis and enhances pancreatic cancer cell exosome uptake. This property enabled efficient delivery of siRNA to the mutant KRAS target. SiRNA decreased KRAS mRNA levels in human pancreatic cancer cells, confirming the efficacy of iExosome uptake [99, 102]. In vivo studies on mice treated with iExosomes demonstrated a reduction in pancreatic tumors; nevertheless, after treatment cessation, tumor growth continued, resulting in an increase in animal survival. The discovery gives light on the therapeutic potential of exosomes, but additional human studies are required to evaluate the efficacy of RNA delivery to tumor locations [99].

According to several studies, the CD47-SIRP pathway influences the innate immune system. Antibodies against CD47 are now utilized to accelerate the phagocytosis of cancer cells [103, 104]. Hu5F9-G4 is an example of a monoclonal antibody used in combination with Rituximab to treat refractory B cell non-Hodgkin lymphoma in phase 1 and 2 clinical trials. If an effective medicine can be personalized to PDAC, the condition may be curable [103].

The root of turmeric is used to produce curcumin. Diaz Osterman et al., studied exosomes containing curcumin as a potential therapy for pancreatic cancer [105]. Curcumin has demonstrated anti-inflammatory and anti-cancer activities in both in vitro and in vivo tests; however, its bioavailability during treatment is poor [105]. Curcumin-treated pancreatic cancer cells produced curcumin-containing exosomes. The results of Hoffman modulation microscopy demonstrated apoptosis and decreased tumor cell sensitivity in pancreatic cancer cells treated with exosomal curcumin, indicating the anti-cancer activity of curcumin and the efficacy of exosomes as drug delivery vehicles [105, 106].

Chemotherapy kills cancer cells by interfering with their capacity to conduct mitosis, thereby halting their fast multiplication [107]. Nevertheless, because the approach is nonselective, it can injure healthy tissue, resulting in a range of negative side effects. Nanocarriers are the most recent development in cancer therapy. The high death rate associated with pancreatic cancer might benefit from advances in treatment and diagnosis [107]. Exosomes are more effective than liposomes, according to several studies. Currently, liposomes are the most prevalent technique of drug administration [47]. A benefit of exosomal therapy is that biomolecules, such as nucleic acids, can circulate throughout the body while retaining their structure for extended periods of time. As a result of their endocytic origin, exosomal membranes contain a range of proteins involved in cellular endocytosis, which increases the delivery of their contents and is advantageous for drug administration [47, 108]. Exosome administration techniques have also been evaluated in clinical trials, with encouraging results. The reduction of exosome secretion has also been investigated as a potential treatment strategy.

Most fibroblasts in PDAC tumors are fibroblasts associated with malignancy (CAFs) [109]. Plans for cancer treatment have concentrated on chemotherapeutics that target epithelial proliferation. Chemoresistance hinders the growth of health. Chemotherapeutic drug exposure produces an increase in cancer-associated fibroblast exosomes expressing the microRNA miR-146a (CAFs) [109–111]. Thereafter, recipient epithelial cells are encouraged to produce the chemo-resistant protein Snail, which enhances cell proliferation and drug resistance. During in vitro investigations, PDAC cells cannot produce drug resistance when CAFs are treated with an exosomal release inhibitor and Gemcitabine, and cancer cell survival is reduced. Myoferlin is a protein with a variety of functions, including receptor-mediated angiogenesis, endocytosis, and muscle regeneration. There is evidence that lung, breast, and pancreatic cancers overexpress myoferlin [112]. Myoferlin expression is lowered in cancerous cells, which results in a decrease in angiogenesis and malignant motility. Hence, inhibiting myoferlin in PADC could be a possible therapeutic target; additional research is required in this area [112].

It is known that tumor-produced exosomes suppress immunosurveillance. According to a study by Lui C et al., the treatment of mice with tumor-secreted exosomes accelerated the development of mouse mammary tumors because natural killer cells, a type of immune system, were less effective at destroying tumor cells. Exosomes generated from tumors may serve as antigens for cancer cells in anti-tumor therapy [113].

**Fig. 4:**
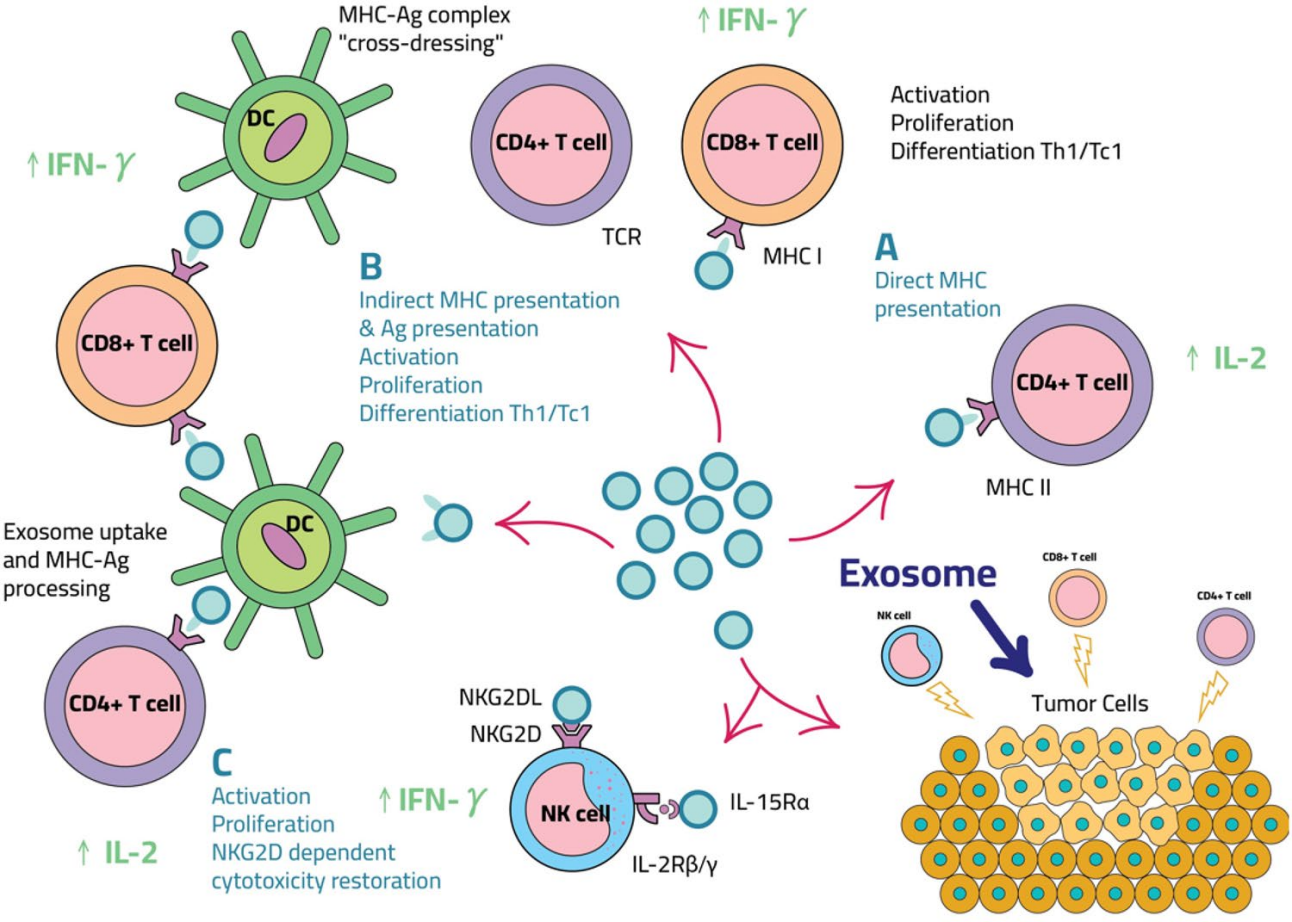
Exosomes in immunotherapy

According to a review by Jonathan P et al., the use of dendritic cell-derived exosomes (DEs) to treat cancer may be more effective [114]. When tumor peptides trigger dendritic cells, dendritic DEs are generated. DEs contain the antigen-presenting components MHC class I and II, as well as the protein CD68, which assists in the efficient priming of *T *cells during antigen presentation [115]. Large concentrations of DEs can excite *T* cells to increase the immune response, and “cross dressing” promotes the transfer of MHC class I peptide to antigen-presenting cells. Patients with lung cancer and melanoma are currently participating in phase 1 clinical trials utilizing DE vaccines [116]. Although no patients with PADC have been included, if the drug’s efficacy is confirmed, it may be used to treat pancreatic cancer [117]. Yet, trial faults, such as a lack of humoral response, prevented considerable T cell activation in certain patients (refer to Fig. 4) [114].

Exosomes can be used in immunotherapy to induce a durable and targeted immune response against cancer cells, in addition to delivering anti-cancer drugs to cancer cells. Exosomes promote DC maturation by exposing them to tumor antigens. Mature DCs can then activate cytotoxic T lymphocytes and natural killer (NK) cells, causing them to attack cancer cells expressing those antigens. T and NK cells are directly stimulated by exosomes. Tumors have an immunosuppressive microenvironment that blocks T and NK cell entry and activation, shielding cancer cells from immune responses. As a result, cancer immunotherapy that employs exosomes to block regulatory *T* cells (TReg cells) and disrupt inhibitory signals to *T* cells appears promising. PDAC may be treated with immunostimulatory drugs and TReg cell inactivation.

In contrast to chemotherapy and liposomal therapy, it is not envisaged that exosomal therapy will induce any physical reactions in patients. Experimenting with a patient's own exosomes as a therapeutic agent could be beneficial and reduce immunological rejection [47].

Exosome-mediated RNAi intra-tumorally verified P21-activated kinase 4 (PAK4) as a therapeutic target in an in vivo PC cancer mouse model. In PC-bearing NSG mice, siPAK4 was tested for tumor development delay and mouse survival. Exo-siPAK4 administered intra-tumorally reduced PC tumor development and improved mouse survival. In siPAK4-treated tumors, H&E staining demonstrated tissue apoptosis [118, 119]. PAK4 knockdown improves PC-bearing mice' survival, suggesting a new PC treatment. In vivo RNAi transfection with PANC-1 Exo was comparable to polyethylenimine (PEI) used as a commercial transfection reagent but safer [118].

Exosomal miR-485-3p released by normal pancreatic ductal epithelial cells into PC cells reduces PC metastasis by directly targeting PAK1. Exosomes or another vehicle could restore miR-485-3p, which would be a novel method of treating PC [120].

Myeloid-derived suppressor cell inhibitors may improve tumor-exosome (TEX)-loaded dendritic cell (DC) immunization (MDSC). UNKC6141 PaCa develops strongly and recover in peripheral blood, bone marrow, lung, and liver after subcutaneous and orthotopic dosing in mice [121]. DC-TEX vaccinations outlasted cytotoxic treatments. Nevertheless, ATRA, Sunitinib, and Gemcitabine (GEM) significantly reduced MDSC, including tumor infiltrating MDSC, tumor cell migration, and metastasis. More activated T cells were retrieved from the tumor and survival time increased with DC-TEX vaccination [122]. ATRA, GEM, and Sun influence MDSC at various maturation and activation phases, boosting DC-TEX vaccination effectiveness. Intrapancreatic tumors were halted [123]. When combined with MDSC maturation and activation inhibitors, TEX-loaded DC immunization may be the best PaCa therapy [121] (See table 1).

**Table 1:** Isolation methods of extracellular vehicles (EVs) [51]

Isolation principle	Method	Sample type	Size (nm)	Advantages	Limitations	References
Buoyant density/Size-selection	Differential centrifugation	Plasma, urine, and cell medium	20–100	Low-cost	Low reproducibility and Impurities Damage of exosomes With Variable efficiency	[52]
	Density gradient centrifugation	Urine and cell medium	20–100	High Purity	Complexity Loss of sample	[53]
	Size-exclusive chromatography	Platelet-free plasma, urine and cell medium	50–500	Reproducibility and exosomes integrity	High cost and complexity	[54]
	Ultrafiltration	Plasma, urine, and Cell medium	50–250	Simplicity and Pure preparation	Loss of sample and exosomes deformation	[15, 55]
	Hydrostatic dialysis	Urine	50–90	Low-cost	Additional purification from bacteria	[56]
Precipitation or phase separation	Precipitation with protamine	Cell culture	–	Low-cost Simplicity and Preservation of EVs integrity	Need purification Long duration	[57]
	Precipitation with polymers	Plasma (PEG) Urine, plasma, or cell medium	50–200	Low cost (PEG) Simplicity EVs integrity	High−cost with high Contamination	[58]
	Precipitation with sodium acetate	Cell culture	–	Low-cost and Simplicity EVs integrity Purity Efficiency	Contamination with non-exosomes protein	[59]
	Precipitation of protein with organic solvent	Plasma	20–300	Low-cost and simplicity	Aggregation	[60]
	Two-phase isolation	Plasma, mixture of exosomes and proteins	Exosomes	Low-cost, Simplicity and purity efficiency	Contamination	[61]
Affinity interaction	Antibodies to EV receptors	Cell medium Plasma	40–150	High purity and selectivity	High cost and exosomes damage	[62]
	Phosphatidylserine binding proteins	Plasma, urine, and cell medium	100	Reversible binding and Simplicity	High cost	[63]
	Heparin-modified sorbents	Plasma and cell medium	–	Low-cost EVs integrity	Need initial purification and ultracentrifugation	[64]
	Binding of heat shock proteins	Plasma, urine, and cell medium	30–100	Exosomes integrity	High cost	[65]
	Lectins	Urine	–	Low-cost, simplicity and high purity	Need initial purification and ultracentrifugation	[66]
Size selection, affinityinteraction	Microfluidic tech neologies	Blood Plasma and cell culture	–	Efficiency	High cost and complexity	[67]

*EVs* extracellular vesicles; *PEG* polyethylene glycol

#### Exosomes and immunity to cancer

The impact of exosomes on cancer is growing in popularity. As can be observed in Table 2, exosomes promote tumor progression and metastasis. As exosomes promote or suppress immunological responses in a variety of malignancies, including pancreatic cancer (Table 2) [124], they may transport immunotherapeutic medicines for the treatment of cancer. Increased NK cell migration and cytolysis are stimulated by Hsp70/Bag-4-rich exosomes released by pancreatic cancer cells [125]. Cancer exosomes, according to the majority of studies, suppress the immune response to Pancreatic ductal adenocarcinoma (PDAC). Higher levels of miR-203 are found in those with PDAC [126]. MiR-203-expressing exosomes from patients with pancreatic cancer inhibit TLR4, TNF, and IL-12, thereby blocking antigen presentation by dendritic cells (DCs) [127]. TLR4 deletion inhibited DC expression of tumor-specific antigens in mice models and human patients with breast cancer. The immune response is maintained when DC TLR4 is produced, increasing the efficacy of radiation and chemotherapy [128]. MiR-203-positive exosomes in pancreatic cancer may represent therapeutic targets. MiR-212-3p inhibited the expression of RFXAP and MHC class II in DCs derived from pancreatic cancer exosomes. PDAC is immune tolerant because DCs are unable to activate CD4 + T cells [129]. Downregulating pancreatic cancer cells or limiting their exosomes increases miR-212-3p and prevents it from reducing the DC antigen-presenting capabilities. TReg cell proliferation was upregulated while CD8 + T cell numbers were downregulated in SMAD4-expressing PDAC cells, as reported by Basso et al. [130]. These results are consistent with those found in the tumor microenvironment of PDAC in mice [131] and humans [132], where there is an increase in TReg cells and a decrease in CD8 + T cell infiltration. Tumor immunological infiltration has a small number of resting CD8 + T lymphocytes [131]. Chen et al., [133] proposed that dysfunction due to infiltration of CD8 + T cells is to responsibility. Several PD-L1-positive exosomes were found in the blood of melanoma patients, as was previously reported by Chen et al., Exosomes released by PD-L1 + melanoma cells suppress CD8 + T lymphocyte expansion and tumor invasion. If these findings are also true for PDAC, they may shed light on the failure of anti-PD-L1 immunotherapy and highlight the need for restricting the production and uptake of exosomes, or may be eliminating them entirely, as an adjuvant cancer treatment. Tumor exosomes from head and neck cancer cell lines inhibit CD8 + T cells, as shown by Maybruck et al., [134]. This inhibits the expansion and activation of responder T cells. T cell invasion into tumor microenvironments is also inhibited by increased Gal-1 expression in PDAC mice [135]. It is possible that PDAC tumor cells, like those of the head and neck, load Gal-1 into exosomes to dampen immunological responses. MDSCs and M2 macrophages predominate in PDAC microenvironments [131, 132, 136]. Tumor growth, lymphangiogenesis, lymphatic metastasis, and a dismal prognosis at the pancreatic cancer invasive front were all sped up by M2 macrophages [132, 136]. AsPC-1 pancreatic cancer cells, which metastasized extensively, were found in the ascites, and their exosomes aggravated macrophage immunosuppression, encouraging the development of pro-tumor macrophages. AsPC-1-derived exosomes promote pancreatic cancer development, metastasis, and angiogenesis by elevating macrophage growth factors and cytokines. Macrophages were induced to produce more prostaglandin E2 after treatment (PGE2). PGE2 inhibits dendritic cell maturation and invasion in kidney cancer [137], as well as CD8 + *T* cell activation. Conditioned medium from lung cancer cells shows that prostaglandin E2 (PGE2) stimulation of TReg cells increases their suppressive function [138]. The effect of pancreatic cancer exosomes on macrophages may contribute to the immunosuppressive PDAC microenvironment. Immunosuppression is maintained in pancreatic cancer by Myeloid-Derived Suppressor Cells (MDSCs) [131, 132]. Wang et al., [139] showed that exosomes released by human pancreatic cancer cells stimulate MDSC growth in vivo. MDSCs have been found invading PDAC [131, 132]. Exosomes released from BxPC3 pancreatic cancer cells had opposing effects on the numbers of MDSCs and DCs in human peripheral blood mononuclear cells. Increased calcium flux and glycolysis were seen in immunosuppressed myeloid cells after exposure to Smad4-deficient BxPC3 exosomes. Glycolytic enzymes secreted by BxPC3-Smad4 + exosomes and hsa-miR-494-3p in myeloid cells regulate calcium flow and glycolysis [130].

**Table 2:** Exosomes impact on cancer

Impact on Cancer	Mechanism	References
Increase tumor growth and progression	Improve tumor growth and proliferation	[143, 144]
	Promote angiogenesis	[145, 146]
	Drug resistance	[109, 147]
	Remodeling of the extracellular matrix	[148, 149]
	Initiate transition of epithelial to mesenchymal cells	[150, 151]
	Reprogram the metabolic system	[152]
Enhance tumor cells' ability to avoid immune response	Initiate large granular lymphocytes (LGL)	[153]
	Stop antigen-presenting cell (APC)	[154, 155]
	Prefer immunosuppressive macrophage phenotypes	[156]
	Promote myeloid cell differentiation into myeloid-derived suppressor cells (MDSCs)	[139]
	Proliferate regulatory T cells	[157]
	Decrease effector T cell proliferation and activation	[133, 158]
Tumor metastasis	Stimulate inflammatory responses at the metastatic site	[78, 140]
	Pre-metastatic niche formation via immune cells suppression	[140, 159]

Cancer exosomes may contribute to the metastatic spread of PDAC [3]. It is possible that exosomes made by tumors can travel to other parts of the body and stimulate tumor cell proliferation there. Exosomal integrins target the liver and lungs to receive pancreatic cancer exosomes [140]. Liver Kupffer macrophages are attracted to exosomes with overexpressed ITGv5, while lung fibroblasts and epithelial cells are attracted to exosomes with overexpressed ITG6/4 and ITG6/1 [140]. Exosomes guide target cells toward metastasis by instructing them, promoting inflammation, and recruiting suppressive immune cells [78, 140]. Pancreatic cancer exosomes generate a tumorigenic environment in the liver and attract cells from bone marrow [78, 141]. Panc02-H7 exosomes promoted MDSC and hepatic myeloid cell infiltration in orthotopic pancreatic cancer rat models. Panc02-H7 exosomes favored Stat3 in myeloid infiltrates [141]. Myeloid cell immunosuppression may facilitate metastasis [142]. The immune tolerance cascade triggered by exosomes from pancreatic cancer at distant sites is, however, poorly understood and warrants further study.

Immunosuppression is seen in the vast majority of exosomes released by pancreatic cancer. Exosomes and the immunosuppressive microenvironment of pancreatic cancer cells are groundbreaking discoveries. According to these studies, cancer exosomes should alert the patient's immune system, which will then attack cancer cells.

## Conclusion

As a result of chemotherapy resistance and the recurrence of tumors, chemotherapy and surgery are no longer as effective in treating pancreatic cancer. Exosomes are extracellular vesicles that facilitate intracellular communication between distant and nearby cells [160]. It has been suggested that exosomes are essential for the maturation of neutrophils, which are required for metastasis. Despite the fact that these pathways have made it feasible to find therapeutic targets, this essay has not discussed their additional activities in angiogenesis, immunological resistance, or cancer [75]. Many clinical trials have utilized exosomes as nanocarriers for immunotherapy and cancer therapy medicines. This sort of treatment offers numerous benefits, including reduced toxicity and an extended half-life [47]. Often recommended treatments for PDAC include chemotherapy and immunosuppression inhibitors, which may improve survival rates due to reduced tumor growth [114, 161]. Exosomes produced by dendritic cells are being studied as immunomodulatory agents in a study with encouraging in vitro and preclinical outcomes [114]. Exosomes have the potential to fundamentally alter cancer treatment, but several issues must be resolved prior to their clinical application. Given that the research is still in its infancy, future years will shed light on a variety of options [47].

## Data Availability

No data was used for the research described in the article.

## References

[1] Qiu J (2018). Extracellular vesicles as mediators of the progression and chemoresistance of pancreatic cancer and their potential clinical applications. Mol Cancer.

[2] Kanji, Z.S. and S. Gallinger, 2013 Diagnosis and management of pancreatic cancer. CMAJ: Canadian Medical Association journal = journal de l'Association medicale canadienne. **185**(14): 1219–1226. 10.1503/cmaj.121368 PMC3787168 23610017

[3] Greenwell M, Rahman PKSM (2015). Medicinal plants: their use in anticancer treatment. Int J Pharm Sci Res.

[4] Pappalardo A (2021). Adjuvant treatment in pancreatic cancer: shaping the future of the curative setting. Front Oncol.

[5] van Roessel S (2020). Evaluation of adjuvant chemotherapy in patients with resected pancreatic cancer after neoadjuvant FOLFIRINOX treatment. JAMA Oncol.

[6] Yachida S (2010). Distant metastasis occurs late during the genetic evolution of pancreatic cancer. Nature.

[7] van den Boogaard WMC, Komninos DSJ, Vermeij WP (2022). Chemotherapy side-effects: not All DNA damage is equal. Cancers.

[8] Principe DR (2021). The current treatment paradigm for pancreatic ductal adenocarcinoma and barriers to therapeutic efficacy. Front Oncol.

[9] Johnstone RM (1987). Vesicle formation during reticulocyte maturation: association of plasma membrane activities with released vesicles (exosomes). J Biol Chem.

[10] Simons M, Raposo G (2009). Exosomes–vesicular carriers for intercellular communication. Curr Opin Cell Biol.

[11] Zlotogorski-Hurvitz A (2015). Human saliva-derived exosomes: comparing methods of isolation. J Histochem Cytochem: Off J Histochem Soc.

[12] Abels ER, Breakefield XO (2016). Introduction to extracellular vesicles: biogenesis, RNA cargo selection, content, release, and uptake. Cell Mol Neurobiol.

[13] Raposo G, Stoorvogel W (2013). Extracellular vesicles: exosomes, microvesicles, and friends. J Cell Biol.

[14] Chen J (2022). Review on strategies and technologies for exosome isolation and purification. Front Bioeng Biotechnol.

[15] Lobb RJ (2015). Optimized exosome isolation protocol for cell culture supernatant and human plasma. J Extracell Vesicles.

[16] Xie F (2019). Extracellular vesicles in cancer immune microenvironment and cancer immunotherapy. Adv Sci (Weinh).

[17] Mimeault M, Batra SK (2014). Molecular biomarkers of cancer stem/progenitor cells associated with progression, metastases, and treatment resistance of aggressive cancers. Cancer Epidemiol Biomark Prev.

[18] Lan B (2019). The role of exosomes in pancreatic cancer. Int J Mol Sci.

[19] Moreno-Gonzalo O, Fernandez-Delgado I, Sanchez-Madrid F (2018). Post-translational add-ons mark the path in exosomal protein sorting. Cell Mol Life Sci.

[20] Li X-X (2023). The roles of exosomal proteins: classification, function, and applications. Int J Mol Sci.

[21] Dai J (2020). Exosomes: key players in cancer and potential therapeutic strategy. Signal Transduct Target Ther.

[22] Schopf FH, Biebl MM, Buchner J (2017). The HSP90 chaperone machinery. Nat Rev Mol Cell Biol.

[23] Tang X (2019). Tumour-secreted Hsp90alpha on external surface of exosomes mediates tumour - stromal cell communication via autocrine and paracrine mechanisms. Sci Rep.

[24] Treiber T, Treiber N, Meister G (2019). Regulation of microRNA biogenesis and its crosstalk with other cellular pathways. Nat Rev Mol Cell Biol.

[25] Yang G (2020). Exosomal miR-21/Let-7a ratio distinguishes non-small cell lung cancer from benign pulmonary diseases. Asia Pac J Clin Oncol.

[26] Huang X (2015). Exosomal miR-1290 and miR-375 as prognostic markers in castration-resistant prostate cancer. Eur Urol.

[27] Shi Y (2020). Exosomal miR-1246 in serum as a potential biomarker for early diagnosis of gastric cancer. Int J Clin Oncol.

[28] Brown TJ, James V (2021). The role of extracellular vesicles in the development of a cancer stem cell microenvironment niche and potential therapeutic targets: a systematic review. Cancers.

[29] Hu C (2020). Role of exosomal microRNAs in lung cancer biology and clinical applications. Cell Prolif.

[30] Grimolizzi F (2017). Exosomal miR-126 as a circulating biomarker in non-small-cell lung cancer regulating cancer progression. Sci Rep.

[31] Wang B, Tan Z, Guan F (2019). Tumor-derived exosomes mediate the instability of cadherins and promote tumor progression. Int J Mol Sci.

[32] Wang J (2014). Bone marrow stromal cell-derived exosomes as communicators in drug resistance in multiple myeloma cells. Blood.

[33] Hu Y (2015). Fibroblast-derived exosomes contribute to chemoresistance through priming cancer stem cells in colorectal cancer. PLoS ONE.

[34] Zhao W (2019). Recent progress in characterizing long noncoding rnas in cancer drug resistance. J Cancer.

[35] Li J (2017). LncRNA-ATB: an indispensable cancer-related long noncoding RNA. Cell Prolif.

[36] Conigliaro A (2015). CD90+ liver cancer cells modulate endothelial cell phenotype through the release of exosomes containing H19 lncRNA. Mol Cancer.

[37] Deng H (2016). Role of long non-coding RNA in tumor drug resistance. Tumour Biol.

[38] Chen QN (2017). Long non-coding RNAs in anti-cancer drug resistance. Oncotarget.

[39] Cheng X (2018). Circular RNA VMA21 protects against intervertebral disc degeneration through targeting miR-200c and X linked inhibitor-of-apoptosis protein. Ann Rheum Dis.

[40] Li J (2018). Circular RNA IARS (circ-IARS) secreted by pancreatic cancer cells and located within exosomes regulates endothelial monolayer permeability to promote tumor metastasis. J Exp Clin Cancer Res.

[41] Chen J (2021). CircRNA ciRS-7: a novel oncogene in multiple cancers. Int J Biol Sci.

[42] Jamal M (2019). Recent Progress on circular RNA Research in acute myeloid leukemia. Front Oncol.

[43] Kristensen LS (2019). The biogenesis, biology and characterization of circular RNAs. Nat Rev Genet.

[44] Smid M (2019). The circular RNome of primary breast cancer. Genome Res.

[45] Li Y (2015). Circular RNA is enriched and stable in exosomes: a promising biomarker for cancer diagnosis. Cell Res.

[46] Chen H (2021). Exosomes, a new star for targeted delivery. Front Cell Develop Biol.

[47] Batista IA, Melo SA (2019). Exosomes and the Future of Immunotherapy in pancreatic cancer. Int J Mol Sci.

[48] Hussen BM (2022). Strategies to overcome the main challenges of the use of exosomes as drug carrier for cancer therapy. Cancer Cell Int.

[49] Yakubovich EI, Polischouk AG, Evtushenko VI (2022). Principles and problems of exosome isolation from biological fluids. Biochem Suppl Series A Membr Cell Biol.

[50] Ahn S-H (2022). Manufacturing therapeutic exosomes: from bench to industry. Mol Cells.

[51] Nicoletti A (2023). Diagnostic and prognostic role of extracellular vesicles in pancreatic cancer: current evidence and future perspectives. Int J Mol Sci.

[52] Livshits MA (2015). Isolation of exosomes by differential centrifugation: Theoretical analysis of a commonly used protocol. Sci Rep.

[53] Greening DW, Posch A (2015). A Protocol for Exosome Isolation and Characterization: Evaluation of Ultracentrifugation, Density-Gradient Separation, and Immunoaffinity Capture Methods. Proteomic Profiling: Methods and Protocols.

[54] Gámez-Valero A (2016). Size-exclusion chromatography-based isolation minimally alters extracellular vesicles’ characteristics compared to precipitating agents. Sci Rep.

[55] Salih M, Zietse R, Hoorn EJ (2014). Urinary extracellular vesicles and the kidney: biomarkers and beyond. Am J Physiol Renal Physiol.

[56] Tataruch-Weinert D (2016). Urinary extracellular vesicles for RNA extraction: optimization of a protocol devoid of prokaryote contamination. J Extracell Vesicles.

[57] Deregibus MC (2016). Charge-based precipitation of extracellular vesicles. Int J Mol Med.

[58] Andreu Z (2016). Comparative analysis of EV isolation procedures for miRNAs detection in serum samples. J Extracell Vesicles.

[59] Brownlee Z (2014). A novel “salting-out” procedure for the isolation of tumor-derived exosomes. J Immunol Methods.

[60] Gallart-Palau X, Serra A, Sze SK (2016). Enrichment of extracellular vesicles from tissues of the central nervous system by PROSPR. Mol Neurodegener.

[61] Shin H (2015). High-yield isolation of extracellular vesicles using aqueous two-phase system. Sci Rep.

[62] Ko J, Carpenter E, Issadore D (2016). Detection and isolation of circulating exosomes and microvesicles for cancer monitoring and diagnostics using micro-/nano-based devices. Analyst.

[63] Nakai W (2016). A novel affinity-based method for the isolation of highly purified extracellular vesicles. Sci Rep.

[64] Balaj L (2015). Heparin affinity purification of extracellular vesicles. Sci Rep.

[65] Knol JC (2016). Peptide-mediated ‘miniprep’ isolation of extracellular vesicles is suitable for high-throughput proteomics. EuPA Open Proteom.

[66] Royo F (2016). Different EV enrichment methods suitable for clinical settings yield different subpopulations of urinary extracellular vesicles from human samples. J Extracell Vesicles.

[67] Caponnetto F (2017). Size-dependent cellular uptake of exosomes. Nanomed Nanotechnol Biol Med.

[68] Hessvik NP, Llorente A (2018). Current knowledge on exosome biogenesis and release. Cell Molecular Life Sci: CMLS.

[69] Zhang J (2015). Exosome and exosomal microRNA: trafficking, sorting, and function. Genom Proteomics Bioinform.

[70] Whiteside TL (2016). Tumor-derived exosomes and their role in cancer progression. Adv Clin Chem.

[71] Kalluri R, LeBleu VS (2020). The biology, function, and biomedical applications of exosomes. Science.

[72] Hannafon BN, Ding W-Q (2013). Intercellular communication by exosome-derived microRNAs in cancer. Int J Mol Sci.

[73] Liu J (2021). The biology, function, and applications of exosomes in cancer. Acta Pharmaceutica Sinica B.

[74] Valadi H (2007). Exosome-mediated transfer of mRNAs and microRNAs is a novel mechanism of genetic exchange between cells. Nat Cell Biol.

[75] Massoumi RL (2019). Emerging evidence for the clinical relevance of pancreatic cancer exosomes. Pancreas.

[76] Akhtar M (2019). Paget's “Seed and Soil” theory of cancer metastasis: an idea whose time has come. Adv Anat Pathol.

[77] Peinado H (2017). Pre-metastatic niches: organ-specific homes for metastases. Nat Rev Cancer.

[78] Costa-Silva B (2015). Pancreatic cancer exosomes initiate pre-metastatic niche formation in the liver. Nat Cell Biol.

[79] Lu P (2011). Extracellular matrix degradation and remodeling in development and disease. Cold Spring Harb Perspect Biol.

[80] Huang L-L, Xia HH-X, Zhu S-L (2014). Ascitic fluid analysis in the differential diagnosis of ascites: focus on cirrhotic ascites. J Clin Transl Hepatol.

[81] van Baal JOAM (2018). Development of peritoneal carcinomatosis in epithelial ovarian cancer: a review. J Histochem Cytochem : Off J Histochem Soc.

[82] Guo Y (2019). Effects of exosomes on pre-metastatic niche formation in tumors. Mol Cancer.

[83] Valenzuela Alvarez M (2019). Metastatic Niches and the modulatory contribution of mesenchymal stem cells and its exosomes. Int J Mol Sci.

[84] Zhang L, Yu D (1871). 2019 Exosomes in cancer development, metastasis, and immunity. Biochimica et Biophysica Acta Rev Cancer.

[85] Heinrichs D (2011). Macrophage migration inhibitory factor (MIF) exerts antifibrotic effects in experimental liver fibrosis via CD74. Proc Natl Acad Sci USA.

[86] Mora Barthelmess R, Stijlemans B, Van Ginderachter JA (2023). Hallmarks of cancer affected by the MIF cytokine family. Cancers.

[87] Funamizu N (2013). Macrophage migration inhibitory factor induces epithelial to mesenchymal transition, enhances tumor aggressiveness and predicts clinical outcome in resected pancreatic ductal adenocarcinoma. Int J Cancer.

[88] Farazi TA (2013). MicroRNAs in human cancer. Adv Exp Med Biol.

[89] Arghiani N, Shah K (2021). Modulating microRNAs in cancer: next-generation therapies. Cancer Biol Med.

[90] Drula R (2020). MicroRNAs from liquid biopsy derived extracellular vesicles: recent advances in detection and characterization methods. Cancers.

[91] Flammang I (2020). Tumor-suppressive miR-192–5p has prognostic value in pancreatic ductal adenocarcinoma. Cancers (Basel).

[92] Reich R (1988). Effects of inhibitors of plasminogen activator, serine proteinases, and collagenase IV on the invasion of basement membranes by metastatic cells. Cancer Res.

[93] Tang S (2013). Sweating the small stuff: microRNAs and genetic changes define pancreatic cancer. Pancreas.

[94] Reese M (2020). Potential of exosomal microRNA-200b as liquid biopsy marker in pancreatic ductal adenocarcinoma. Cancers.

[95] Machida T (2016). miR-1246 and miR-4644 in salivary exosome as potential biomarkers for pancreatobiliary tract cancer. Oncol Rep.

[96] Que R (2013). Analysis of serum exosomal microRNAs and clinicopathologic features of patients with pancreatic adenocarcinoma. World J Surg Oncol.

[97] He C (2021). Comparative recurrence analysis of pancreatic adenocarcinoma after resection. J Oncol.

[98] Jiang Z (2022). Functions and clinical applications of exosomes in pancreatic cancer. Mol Biol Rep.

[99] Kamerkar S (2017). Exosomes facilitate therapeutic targeting of oncogenic KRAS in pancreatic cancer. Nature.

[100] Zhao H, Song S, Ma J, Yan Z, Xie H, Feng Y, Che S (2022). CD47 as a promising therapeutic target in oncology. Front
Immunol.

[101] Luo X (2023). Blocking CD47-SIRPα signal axis as promising immunotherapy in ovarian cancer. Cancer Control: J Moffitt Cancer Center.

[102] Strand MS (2019). Precision delivery of RAS-inhibiting siRNA to KRAS driven cancer via peptide-based nanoparticles. Oncotarget.

[103] Advani R (2018). CD47 Blockade by Hu5F9-G4 and Rituximab in Non-Hodgkin's Lymphoma. N Engl J Med.

[104] Pierpont TM, Limper CB, Richards KL (2018). Past, present, and future of rituximab—the world’s first oncology monoclonal antibody therapy. Front Oncol.

[105] Osterman CJ (2015). Curcumin modulates pancreatic adenocarcinoma cell-derived exosomal function. PLoS ONE.

[106] Osterman CJD (2015). Curcumin modulates pancreatic adenocarcinoma cell-derived exosomal function. PLoS ONE.

[107] Johnsen KB (1846). 2014 A comprehensive overview of exosomes as drug delivery vehicles — Endogenous nanocarriers for targeted cancer therapy. Biochimica et Biophysica Acta Rev Cancer.

[108] Ha D, Yang N, Nadithe V (2016). Exosomes as therapeutic drug carriers and delivery vehicles across biological membranes: current perspectives and future challenges. Acta Pharmaceutica Sinica B.

[109] Richards KE (2017). Cancer-associated fibroblast exosomes regulate survival and proliferation of pancreatic cancer cells. Oncogene.

[110] Li J (2021). The emerging role of exosomes in cancer chemoresistance. Frontiers in Cell and Develop Biol.

[111] Hu S (2022). Natural products exert anti-tumor effects by regulating exosomal ncRNA. Front Oncol.

[112] Blomme A (2016). Myoferlin is a novel exosomal protein and functional regulator of cancer-derived exosomes. Oncotarget.

[113] Liu C (2006). Murine mammary carcinoma exosomes promote tumor growth by suppression of NK cell function. J Immunol.

[114] Pitt JM (2014). Dendritic cell-derived exosomes as immunotherapies in the fight against cancer. J Immunol.

[115] ten Broeke T, Wubbolts R, Stoorvogel W (2013). MHC class II antigen presentation by dendritic cells regulated through endosomal sorting. Cold Spring Harb Perspect Biol.

[116] Hough KP (2017). Exosomes in immunoregulation of chronic lung diseases. Allergy.

[117] Di Marco M (2016). State of the art biological therapies in pancreatic cancer. World J Gastrointest Oncol.

[118] Xu L (2021). Exosome-mediated RNAi of PAK4 prolongs survival of pancreatic cancer mouse model after loco-regional treatment. Biomaterials.

[119] Wang K (2018). P21-activated kinase signalling in pancreatic cancer: new insights into tumour biology and immune modulation. World J Gastroenterol.

[120] Li M (2022). Exosomal miR-485-3p derived from pancreatic ductal epithelial cells inhibits pancreatic cancer metastasis through targeting PAK1. Chin Med J (Engl).

[121] Xiao L (2017). Efficacy of vaccination with tumor-exosome loaded dendritic cells combined with cytotoxic drug treatment in pancreatic cancer. Oncoimmunology.

[122] Finke J (2011). MDSC as a mechanism of tumor escape from sunitinib mediated anti-angiogenic therapy. Int Immunopharmacol.

[123] Xiao L (2017). Efficacy of vaccination with tumor-exosome loaded dendritic cells combined with cytotoxic drug treatment in pancreatic cancer. Oncoimmunology.

[124] Ruivo CF (2017). The biology of cancer exosomes: insights and new perspectives. Cancer Res.

[125] Gastpar R (2005). Heat shock protein 70 surface-positive tumor exosomes stimulate migratory and cytolytic activity of natural killer cells. Cancer Res.

[126] Bloomston M (2007). MicroRNA expression patterns to differentiate pancreatic adenocarcinoma from normal pancreas and chronic pancreatitis. JAMA.

[127] Zhou M (2014). Pancreatic cancer derived exosomes regulate the expression of TLR4 in dendritic cells via miR-203. Cell Immunol.

[128] Apetoh L (2007). Toll-like receptor 4-dependent contribution of the immune system to anticancer chemotherapy and radiotherapy. Nat Med.

[129] Ding G (2015). Pancreatic cancer-derived exosomes transfer miRNAs to dendritic cells and inhibit RFXAP expression via miR-212-3p. Oncotarget.

[130] Basso D (2017). PDAC-derived exosomes enrich the microenvironment in MDSCs in a SMAD4-dependent manner through a new calcium related axis. Oncotarget.

[131] Clark CE (2007). Dynamics of the immune reaction to pancreatic cancer from inception to invasion. Cancer Res.

[132] Ino Y (2013). Immune cell infiltration as an indicator of the immune microenvironment of pancreatic cancer. Br J Cancer.

[133] Chen G (2018). Exosomal PD-L1 contributes to immunosuppression and is associated with anti-PD-1 response. Nature.

[134] Maybruck BT (2017). Tumor-derived exosomes induce CD8(+) T cell suppressors. J Immunother Cancer.

[135] Martinez-Bosch N (2014). Galectin-1 drives pancreatic carcinogenesis through stroma remodeling and Hedgehog signaling activation. Cancer Res.

[136] Kurahara H (2011). Significance of M2-polarized tumor-associated macrophage in pancreatic cancer. J Surg Res.

[137] Ahmadi M, Emery DC, Morgan DJ (2008). Prevention of both direct and cross-priming of antitumor CD8+ T-cell responses following overproduction of prostaglandin E2 by tumor cells in vivo. Cancer Res.

[138] Baratelli F (2005). Prostaglandin E2 induces FOXP3 gene expression and T regulatory cell function in human CD4+ T cells. J Immunol.

[139] Wang Z (2016). CD44v6-competent tumor exosomes promote motility, invasion and cancer-initiating cell marker expression in pancreatic and colorectal cancer cells. Oncotarget.

[140] Hoshino A (2015). Tumour exosome integrins determine organotropic metastasis. Nature.

[141] Yu Z (2017). Pancreatic cancer-derived exosomes promote tumor metastasis and liver pre-metastatic niche formation. Oncotarget.

[142] Chalmin F (2010). Membrane-associated Hsp72 from tumor-derived exosomes mediates STAT3-dependent immunosuppressive function of mouse and human myeloid-derived suppressor cells. J Clin Invest.

[143] Melo SA (2014). Cancer exosomes perform cell-independent microRNA biogenesis and promote tumorigenesis. Cancer Cell.

[144] Beloribi-Djefaflia S, Siret C, Lombardo D (2015). Exosomal lipids induce human pancreatic tumoral MiaPaCa-2 cells resistance through the CXCR4-SDF-1α signaling axis. Oncoscience.

[145] Kucharzewska P (2013). Exosomes reflect the hypoxic status of glioma cells and mediate hypoxia-dependent activation of vascular cells during tumor development. Proc Natl Acad Sci.

[146] Nazarenko I (2010). Cell surface tetraspanin Tspan8 contributes to molecular pathways of exosome-induced endothelial cell activation. Can Res.

[147] Binenbaum Y (2018). Transfer of miRNA in macrophage-derived exosomes induces drug resistance in pancreatic adenocarcinoma. Can Res.

[148] Hendrix A (2010). Effect of the secretory small GTPase Rab27B on breast cancer growth, invasion, and metastasis. J Natl Cancer Inst.

[149] Sidhu SS (2004). The microvesicle as a vehicle for EMMPRIN in tumor–stromal interactions. Oncogene.

[150] Li Z (2018). Tumor-derived exosomal lnc-Sox2ot promotes EMT and stemness by acting as a ceRNA in pancreatic ductal adenocarcinoma. Oncogene.

[151] Blackwell RH, Foreman KE, Gupta GN (2017). The role of cancer-derived exosomes in tumorigenicity & epithelial-to-mesenchymal transition. Cancers.

[152] Wang L (2017). Exosomes derived from pancreatic cancer cells induce insulin resistance in C2C12 myotube cells through the PI3K/Akt/FoxO1 pathway. Sci Rep.

[153] Gastpar R (2005). Heat Shock protein 70 surface-positive tumor exosomes stimulate migratory and cytolytic activity of natural killer cells. Can Res.

[154] Zhou M (2014). Pancreatic cancer derived exosomes regulate the expression of TLR4 in dendritic cells via miR-203. Cell Immunol.

[155] Ding G (2015). Pancreatic cancer-derived exosomes transfer miRNAs to dendritic cells and inhibit RFXAP expression via miR-212–3p. Oncotarget.

[156] Linton SS (2018). Tumor-promoting effects of pancreatic cancer cell exosomes on THP-1-derived macrophages. PLoS ONE.

[157] Basso D (2017). PDAC-derived exosomes enrich the microenvironment in MDSCs in a SMAD4 -dependent manner through a new calcium related axis. Oncotarget.

[158] Martínez-Bosch N (2014). Galectin-1 drives pancreatic carcinogenesis through stroma remodeling and hedgehog signaling activation. Can Res.

[159] Chalmin F (2010). Membrane-associated Hsp72 from tumor-derived exosomes mediates STAT3-dependent immunosuppressive function of mouse and human myeloid-derived suppressor cells. J Clin Investig.

[160] Zhang J (2015). 2015 exosome and exosomal microRNA: trafficking, sorting, and function. Genom Proteomics & Bioinform.

[161] Romagnoli GG (2015). Dendritic cell-derived exosomes may be a tool for cancer immunotherapy by converting tumor cells into immunogenic targets. Front Immunol.

